# Is the Medial Prefrontal Cortex Necessary for Theory of Mind?

**DOI:** 10.1371/journal.pone.0135912

**Published:** 2015-08-24

**Authors:** Alexander Otti, Afra M. Wohlschlaeger, Michael Noll-Hussong

**Affiliations:** 1 Klinik fuer Psychosomatische Medizin und Psychotherapie, Klinikum rechts der Isar, Technische Universitaet Muenchen, Munich, Germany; 2 Abteilung fuer Neuroradiologie, Klinikum rechts der Isar, Technische Universitaet Muenchen, Munich, Germany; 3 Klinik fuer Psychosomatische Medizin und Psychotherapie, Universitaetsklinikum Ulm, Ulm, Germany; UCLA, UNITED STATES

## Abstract

**Background:**

Successful social interaction relies on the ability to attribute mental states to other people. Previous functional neuroimaging studies have shown that this process, described as Theory of Mind (ToM) or mentalization, is reliably associated with activation of the medial prefrontal cortex (mPFC). However, this study presents a novel and surprising finding that provides new insight into the role of the mPFC in mentalization tasks.

**Methodology/Principal Findings:**

Twenty healthy individuals were recruited from a wide range of ages and social backgrounds. Participants underwent functional magnetic resonance imaging (fMRI) while viewing a well-established ToM visual paradigm involving moving triangles. Functional MRI data were analyzed using a classical general linear model. No activation was detected in the medial prefrontal cortex (mPFC) during movement patterns that typically elicit ToM. However, increased activity was observed in the right middle occipital gyrus, right temporoparietal junction (TPJ), left middle occipital gyrus and right inferior frontal gyrus. No correlation was found between participants’ age and BOLD response.

**Conclusions/Significance:**

In contrast with previous neuroimaging research, our findings support the notion that mPFC function is not critical for reasoning about the mental states of others; furthermore, our data indicate that the right TPJ and right inferior frontal gyrus are able to perform mentalization without any contributions from the mPFC.

## Introduction

To achieve successful social interaction, human beings must be able to think of other conspecifics as psychological agents whose behavior is driven by mental states. The process by which one attributes thoughts, intentions and beliefs to oneself and others is described as Theory of Mind (ToM) [1] or mentalization [2]. As demonstrated by Heider and Simmel 70 years ago, this process is fundamentally imprinted in the human brain [3]. In response to simple animated stimuli (e.g., two triangles and a circle that move against or around each other and/or a rectangle), nearly all healthy individuals automatically perceive intentionality and attribute mental states to other entities (e.g., the moving shapes).

ToM tasks activate a large-scale brain network that consists of cortical midline structures such as the medial prefrontal cortex (mPFC) and the precuneus, as well as lateral parietal regions such as the posterior temporal sulcus (i.e., the temporoparietal junction, TPJ) and the temporal pole [4–9]. The mPFC has been specifically identified as being a crucial node within the ToM network [2, 10–12]. However, it is controversial to assume that mPFC activity is necessary for ToM. For instance, one study reported a single case of extensive frontal lobe damage that did not affect the patient’s ToM ability [13]. However, it remains unclear whether this finding can be generalized to other populations.

Considering the body of previous research, this study analyzed a group of healthy individuals who were originally recruited as controls for a naturalistic fMRI study that assessed social understanding and mPFC function in pain disorder. The current study led to a surprising result: mPFC activation was not detected during a ToM task.

## Methods

### Participants

Twenty healthy right-handed, German-speaking individuals of Caucasian ethnicity [14] were recruited from a broad range of ages (7 male, 13 female; mean-age 45.6 years, SD = 14.01, range 24–64 years) and educational backgrounds. Participants gave written informed consent in accordance with the guidelines of the Declaration of Helsinki. Most of the subjects were participants (“control group”) of other neuroimaging studies performed by our research group [15–19]. This study was approved by the Ethics Committee of the Rechts der Isar Hospital, Technical University of Munich. All participants were screened by trained physicians to rule out any neurological or psychiatric disorders.

### Stimuli

The eight silent visual animations that were used in this experiment have been validated by previous functional imaging studies [20–24]. In each animation, two shapes, a small blue triangle and a large red triangle, moved against a white background. Two types of animations were depicted, which represent two different types of social interaction; there were a total of four ToM movies and four random movement movies (R) used in this experiment. The ToM sequence portrayed the triangles coaxing, mocking, seducing and surprising each other [21]. The R sequence was used as a control condition and portrayed the triangles moving or rotating around the background without any intentional interaction. For this study, all of the videos were cropped to the same length (36 sec), leaving the relevant content untouched. A personal computer running the ´Presentation´ software (Neurobehavioural Systems, http://www.neurobs.com) projected stimuli onto a screen utilizing a projector and mirror mount.

### Scanning method and procedure

First, participants completed a training session before the fMRI scan was performed, to familiarize themselves with the stimuli and scanning procedure. During this training session, two videos were presented in a randomized order (one from each of the two conditions); these videos were not utilized during the fMRI session.

During the fMRI scanning session, 8 video stimuli (4 from each category) were presented in a block design. Each stimulus appeared only once during the entire task and represented one block. Each block was followed by a blank screen that was of the same length as the baseline condition, resulting in a total scanning time of 576 sec (8 blocks and 8 baseline conditions at 36 sec each). For each participant, videos were drawn in random order from a library of 8 videos.

Echo planar imaging (EPI) was acquired using a 3T Philips Achieva Scanner (Philips Medical Systems) fitted with a standard 8-channel SENSE head coil. A total of 32 contiguous slices (i.e., no gap), with steep angulation to exclude the eyes, were acquired using a gradient echo EPI sequence utilizing the following parameters: 2000 ms TR; 35 ms TE; 82 degree flip angle; 220 mm FOV; 4 mm slice thickness; 80x80 matrix; voxel size 2.75x2.75 mm; SENSE factor 2. Structural images were obtained using a T1-weighted turbo gradient echo sequence utilizing the following parameters: 9 ms TR; 4 ms TE; 8 degree flip angle; 240 mm FOV; 240x240 matrix; voxel size 1 mm isotropic; 170 slices; no gap.

Immediately after the fMRI session, participants underwent a debriefing session, during which the stimuli were shown again in the same order. After each video clip, each participant was asked a standardized open-ended question ("What happened?") and allowed to provide his/her response without any time restrictions. The answers were recorded, transcribed, rated, and analyzed by averaging the ratings of two independent, trained medical scientists. Ratings were performed according to the original ToM paradigm, using an established scale ranging from 1 to 5 to measure the grade of intentionality attributed to the stimuli [21] (Table 1).

**Table 1:** Results of the post-scan-interview.

	Maximum	ToM	R
	core	mean (sd)	mean (sd)
**Intentionality**	20	14.05 (1.64)	3.85 (2.66)
**Appropriateness**	12	9.55 (1.43)	10.5 (2.12)

### Data analysis

Statistical parametric mapping (SPM5, Wellcome Department of Imaging Neuroscience, London) was utilized for the realignment and coregistration of the EPI-images into T1-weighted images. After transforming the data onto the anatomical space of a template brain (Montreal Neurological Institute), the normalized images were smoothed using an 8 mm FWHM Gaussian kernel.

The first level of our analysis was a subject-wise computation using the functional general linear model. Then, a comparison of the estimates by means of linear contrasts was performed for each animation to determine the specific effects of the different conditions. The resulting set of voxels constituted a statistical parametric map of the t-statistic. The following contrast was computed: “ToM > R”, “ToM > Baseline”, and “R > Baseline”. Correlation analyses were performed between the imaging data and the results of the post-scan intentionality ratings.

Furthermore, a Bayesian analysis was performed to test whether or not the null-hypothesis—i. e.no activation in the mPFC–can be accepted [25]. Given the changes in the regional cerebral blood flow illustrated by Castelli et al. [21], where responses in an mPFC region were similar to that in the superior temporal sulcus, the “theory” to evualuate using Bayes factors is that the response in the mPFC is the same as the response in the right superior temporal gyrus region (which showed a significant “ToM > R”-activity). The following volumes of interest in the mPFC were defined based on peaks (Talairach-Coordinates) found in previous studies (small volume of interest analysis, radius = 15 mm): 12 63 17 and 10 62 30 [22], 14 63 13 and 4 50 29 [23], and -6 58 32 [21]. Furthermore, a volume of interest in the right posterior superior temporal gyrus was defined based on the peak in our study (MNI-Coordinates 54–48 16). The mean BOLD signal change within the volumes of interests was calculated. For the Bayesian analysis, a normal distribution was presumed.

### Statistical thresholds

For the whole-brain analysis, we applied an *a priori* threshold of p < 0.001 for uncorrected data at the voxel level and a threshold of p < 0.05 when data were corrected for family-wise-errors at the cluster level (cluster-extent threshold > 1 voxel). For the correlation-analyses, we used an *a priori* threshold of p < 0.01 for uncorrected data at the voxel level and a threshold of p < 0.05 when data were corrected for family-wise-errors at the cluster level (cluster-extent threshold > 1 voxel). For the Bayesian analysis, Bayes-Factors < 1/3 give substantial evidence for the null-hypothesis over the alternate hypothesis [25].

## Results

**Fig 1:**
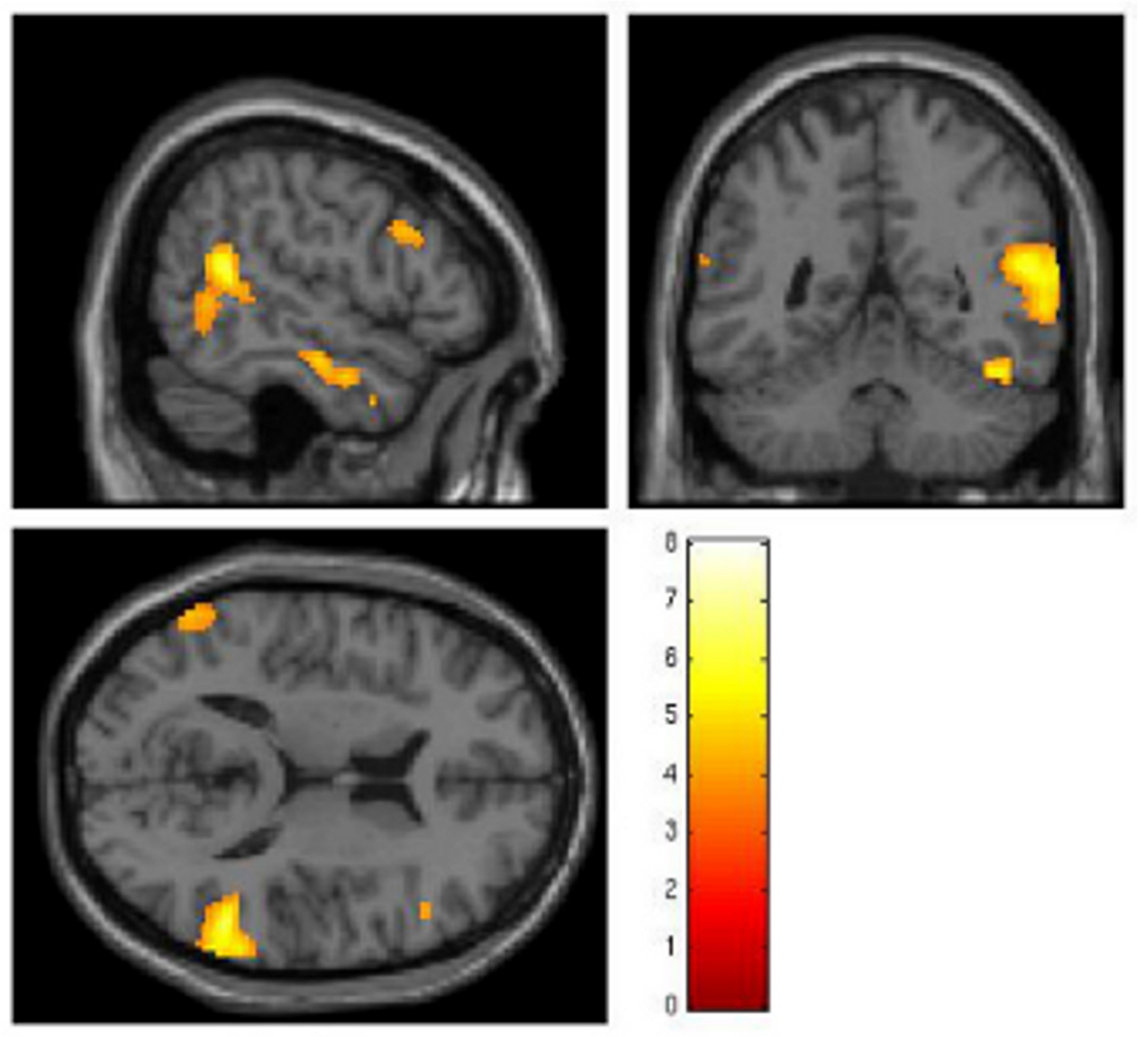
Activations during the “ToM > R” analysis (whole-brain analysis; *a priori* p < 0.001 for uncorrected data at the voxel level and p < 0.05 when data were corrected for family-wise-errors at the cluster level; cluster-extent threshold > 1 voxel; * = also significant at p < 0.05 when data were corrected for family-wise-errors at the voxel level; color bars represent T-values).

Our comparison of the ToM versus R conditions showed a significant signal increase in the right middle occipital gyrus, right superior temporal gyrus, left middle occipital gyrus and right inferior frontal gyrus (Fig 1 and Table 2).

**Table 2:** Activations during the “ToM > R” analysis (whole-brain analysis; *a priori* p < 0.001 for uncorrected data at the voxel level and p < 0.05 when data were corrected for family-wise-errors at the cluster level; cluster-extent threshold > 1 voxel; * = also significant at p < 0.05 when data were corrected for family-wise-errors at the voxel level).

Region	MNI	k	T	p at (cluster level)
R middle occipital gyrus*	28–94 4	702	8.01	0.00
*R superior temporal gyrus*	54–48 16	1248	6.30	0.00
*L middle occipital gyrus*	-34–94–8	212	5.52	0.01
*L middle occipital gyrus*	-60–64 4	358	5.17	0.002
*R inferior frontal gyrus*	46 12 18	182	4.88	0.022

No significant activation was seen in the mPFC, even at the more lenient threshold of p < 0.01 (uncorrected at the voxel level).

No significant correlation was seen between the imaging results and the post-can intentionality ratings.

The comparison of the ToM versus Baseline conditions showed a significant signal increase in the right inferior occipital gyrus, left inferior frontal gyrus, right thalamus, right inferior frontal gyrus, and left superior temporal gyrus (S1 Fig and S1 Table). The comparison of the R versus Baseline conditions showed a significant signal increase in the left superior parietal lobule, left superior frontal gyrus, left thalamus, and right middle frontal gyrus (S2 Fig and S2 Table). The Bayesian analysis provided substantial evidence (Bayes-Factor < 1/3) that “ToM > R” does not elicit significant activation [25] (Table 3).

**Table 3:** Bayesian analysis to evaluate the theory that the response in the mPFC is the same as the response in the right superior temporal gyrus during “ToM > R” (volume of interest (VOI): mPFC-coordinates based on literature, right posterior superior temporal gyrus coordinates based on our sample (MNI: 54–48 16), radius 15 mm; mean and standard deviation (SD) represent the average BOLD-signal within a volume of interest).

mPFC					R post. sup. temporal gyrus			Bayes-factor
VOI (Talairach)	VOI (MNI)	Reference	mean	SD	VOI (MNI)	mean	SD	
12 63 17	12 64 22	[22]	0.0327	0.3143	54–46 16	0.2564	0.2177	**0.20**
10 62 30	10 65 27	[22]	0.0366	0.3420	54–46 16	0.2564	0.2177	**0.24**
14 63 13	14 64 18	[23]	0.0269	0.2829	54–46 16	0.2564	0.2177	**0.18**
4 50 29	4 50 34	[23]	0.0375	0.3323	54–46 16	0.2564	0.2177	**0.23**
-6 58 32	-6 58 38	[21]	0.0172	0.3406	54–46 16	0.2564	0.2177	**0.20**

## Discussion

In this study, we examined the neural representations of ToM in healthy participants using a well-established fMRI paradigm.

In contrast to the vast majority of imaging studies on ToM (for review see [4, 5–7]), mPFC activation was not detected in our study. Furthermore, our findings expand on the results of recent studies on brain lesions and present an additional piece of evidence to refute the assumption that mPFC activity is essential for mentalization in adults [13, 26].

There is converging evidence supporting the notion that, on a functional level, the mPFC is not ToM-specific but domain-general [27] because this region has been implicated in various psychological processes (e.g., default mode activity, autobiographic memory, self-reflection, prospection, and predicting preferences), personality traits, emotional empathy and affective regulation [17, 28–40]. For example, the ventral mPFC contributes to emotional ToM [41] in terms of empathic concern for another person’s affective state [17, 42–46], whereas the dorsal mPFC mediates “triadic attention” and collaboration [47] (i.e., when two persons pay attention to the same object [48, 49]). MPFC function can be synoptically described as the neural basis of self-referential thinking, a process that typically arises when an environmental stimulus is specifically relevant to one’s own tasks. However, the mere observation of two social agents interacting with each other may not necessarily be personally salient to an observer and does not automatically require self-related processes such as joint attention or emotional concern [50]. Our findings support the notion that mPFC activity is not involved in mentalization itself; rather, it contributes to the self-related processes that are often associated with ToM.

Moreover, one may speculate that the mPFC is crucial only for the acquisition of ToM during childhood and adolescence, because it does not appear to be relevant for mentalization during adulthood. In this context, mPFC activation has been shown to shift from the ventral to dorsal region during maturation, whereas no age-related changes were found in the TPJ and temporal poles [23]. Furthermore, there appears to be a developmental trajectory from higher to lower activation in the mPFC and adjacent paracingulate gyrus [51, 52]. Although research on mentalization during normal aging or psychogenesis is still in its infancy, the existing results suggest an immense plasticity of ToM functions, which can likely be adopted by other areas of the brain besides than the mPFC.

In agreement with previous research [4–6, 8], our study found activation within the right TPJ and inferior frontal gyrus. Unlike the mPFC, there is converging evidence to support the notion that the right TPJ is a critical region for ToM [53–56]. This region supports various cognitive tasks, including ToM, empathy, the perception of agency, and attentional reorientation to salient stimuli [57–60]. However, it is unclear if a common psychobiological mechanism (involved in both attention and ToM) exists and is performed by this region or if mentalization relies on a distinct module within the posterior region of the right TPJ, as suggested by Scholz et al. (2009) and Young et al. [61]. Our study also found activation in the right inferior frontal gyrus, which is a region that has been reliably associated with social cognition (for review see [5]). As with the right TPJ, the right inferior frontal gyrus is activated when salient cues are detected and is also involved in attentional reorientation [57]. The TPJ and inferior frontal gyrus appear to be sufficient for ToM without contribution from the mPFC; this finding highlights the pivotal role of these regions in mentalization.

In summary, our report indicates that mPFC function is neither selective nor necessary for cognitive ToM [62]. However, further direct experimental imaging studies will be crucial to explore whether a ToM-related neural network exists, in which the mPFC, TPJ and inferior frontal gyrus can be flexibly integrated, or whether an innate ToM module specifically instantiates reasoning about the mental state of others.

## Supporting Information

S1 Fig Activations during the “ToM > Baseline” analysis (whole-brain analysis; *a priori* p < 0.001 for uncorrected data at the voxel level and p < 0.05 when data were corrected for family-wise-errors at the cluster level; cluster-extent threshold > 1 voxel). (TIF)

S2 Fig Activations during the “ToM > R” analysis (whole-brain analysis; *a priori* p < 0.001 for uncorrected data at the voxel level and p < 0.05 when data were corrected for family-wise-errors at the cluster level; cluster-extent threshold > 1 voxel). (TIF)

S1 Table Activations during the “ToM > Baseline” analysis (whole-brain analysis; *a priori* p < 0.001 for uncorrected data at the voxel level and p < 0.05 when data were corrected for family-wise-errors at the cluster level; cluster-extent threshold > 1 voxel). (DOCX)

S2 Table Activations during the “R > Baseline” analysis (whole-brain analysis; *a priori* p < 0.001 for uncorrected data at the voxel level and p < 0.05 when data were corrected for family-wise-errors at the cluster level; cluster-extent threshold > 1 voxel). (DOCX)
