# Supplementary figures and images for: Is the Medial Prefrontal Cortex Necessary for Theory of Mind?
Source: PLoS One. 2015 Aug 24;10(8):e0135912. doi: 10.1371/journal.pone.0135912 (PMC4547759; doi:10.1371/journal.pone.0135912)

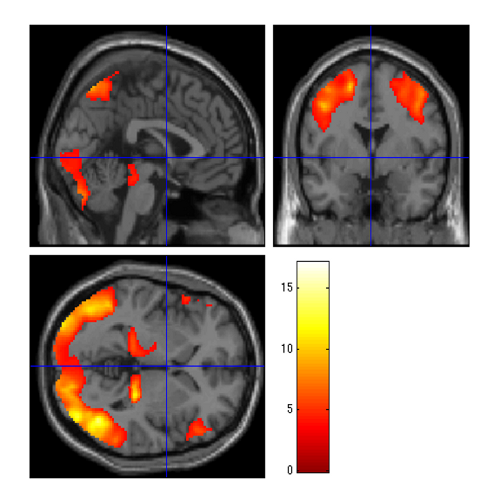

Supplement: S1 Fig — (TIF) [file pone.0135912.s001.tif]

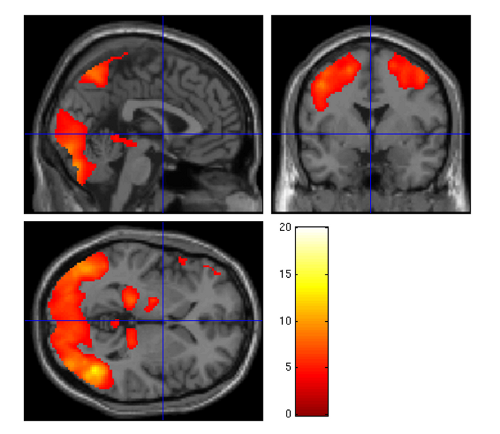

Supplement: S2 Fig — (TIF) [file pone.0135912.s002.tif]
