# Supplementary material for: Is the Medial Prefrontal Cortex Necessary for Theory of Mind?
Source: PLoS One. 2015 Aug 24;10(8):e0135912. doi: 10.1371/journal.pone.0135912 (PMC4547759; doi:10.1371/journal.pone.0135912)
Supplement: S1 Table — (DOCX) [file pone.0135912.s003.docx]

| **Region** | **MNI** | **k** | **T** | **p**  **at (cluster level)** |
| --- | --- | --- | --- | --- |
| *R inferior occipital gyrus* | 44 -80 -12 | 33824 | 17.08 | 0.00 |
| *L inferior frontal gyrus* | -46 8 36 | 3924 | 13.16 | 0.00 |
| *R thalamus* | 24 -30 2 | 2337 | 11.37 | 0.00 |
| *R inferior frontal gyrus* | 62 22 18 | 4102 | 9.12 | 0.00 |
| *L superior temporal gyrus* | -42 22 -24 | 150 | 5.64 | 0.039 |
