# Supplementary material for: Is the Medial Prefrontal Cortex Necessary for Theory of Mind?
Source: PLoS One. 2015 Aug 24;10(8):e0135912. doi: 10.1371/journal.pone.0135912 (PMC4547759; doi:10.1371/journal.pone.0135912)
Supplement: S2 Table — (DOCX) [file pone.0135912.s004.docx]

| **Region** | **MNI** | **k** | **T** | **p**  **at (cluster level)** |
| --- | --- | --- | --- | --- |
| *L superior parietal lobule* | -16 -7 54 | 40263 | 20.00 | 0.00 |
| *L superior frontal gyrus* | -20 -4 60 | 3781 | 9.33 | 0.00 |
| *L thalamus* | -16 -30 8 | 2065 | 8.14 | 0.00 |
| *R middle frontal gyrus* | 46 0 52 | 3673 | 6.55 | 0.00 |
